# A Dynamic Surface Gateway Placement Scheme for Mobile Underwater Networks †

**DOI:** 10.3390/s19091993

**Published:** 2019-04-28

**Authors:** Jun Liu, Wenxue Guan, Guangjie Han, Jun-Hong Cui, Lance Fiondella, Manal Al-Bzoor

**Affiliations:** 1 College of Computer Science and Technology, Jilin University, Changchun 130012, China; liujun1509@jlu.edu.cn (J.L.); guanwx18@mails.jlu.edu.cn (W.G.); junhong_cui@jlu.edu.cn (J.-H.C.); 2 State Key Laboratory of Robotics, Shenyang Institute of Automation, Chinese Academy of Sciences, Shenyang 110016, China; 3 College of Engineering, Nanjing Agricultural University, Nanjing 210095, China; 4 College of Internet of Things Engineering, Hohai University, 200 North Jinling Road, Changzhou 213022, China; 5 State Key Laboratory of Acoustics, Institute of Acoustics, Chinese Academy of Sciences, Beijing 100190, China; 6 Department of Electrical and Computer Engineering (ECE), University of Massachusetts, Dartmouth, MA 02747, USA; lfiondella@umassd.edu; 7 Department of Computer Engineering, Yarmouk University, Irbid 21163, Jordan; mbzoor@yu.edu.jo

**Keywords:** underwater wireless sensor networks (UWSNs), dynamic surface gateway placement, optimization

## Abstract

Deployment of surface-level gateways holds potential as an effective method to alleviate high-propagation delays and high-error probability in an underwater wireless sensor network (UWSN). This promise comes from reducing distances to underwater nodes and using radio waves to forward information to a control station. In an UWSN, a dynamic energy efficient surface-level gateway deployment is required to cope with the mobility of underwater nodes while considering the remote and three-dimensional nature of marine space. In general, deployment problems are usually modeled as an optimization problem to satisfy multiple constraints given a set of parameters. One previously published static deployment optimization framework makes assumptions about network workload, routing, medium access control performance, and node mobility. However, in real underwater environments, all these parameters are dynamic. Therefore, the accuracy of performance estimates calculated through static UWSN deployment optimization framework tends to be limited by nature. This paper presents the *Prediction-Assisted Dynamic Surface Gateway Placement* (PADP) algorithm to maximize the coverage and minimize the average end-to-end delay of a mobile underwater sensor network over a specified period. PADP implements the Interacting Multiple Model (IMM) tracking scheme to predict the positions of sensor nodes. The deployment is determined based on both current and predicted positions of sensor nodes, which enables better coverage and shorter end-to-end delay. PADP uses a branch-and-cut approach to solve the optimization problem efficiently, and employs a disjoint-set data structure to ensure connectivity. Simulation results illustrate that PADP significantly outperforms a static gateway deployment scheme.

## 1. Introduction

Over recent years, underwater sensor networks (UWSNs) have attracted considerable attention from academia and industry [1,2,3,4,5,6] allowing a wide range of marine applications, including coastal surveillance, environmental monitoring, undersea exploration, disaster prevention, and mine reconnaissance. However, due to the high attenuation of radio waves in water, acoustic waves were chosen by UWSNs as the best solution for underwater communication. On the other hand, underwater acoustic communications and networking imply unique and challenging characteristics, such as low bandwidth, long propagation delay, high-error probability, and sensor node mobility (passive or proactive) found in mobile networks. These challenges should be considered for each layer of the network protocol stack [7,8,9,10,11,12].

In an UWSN, sensor nodes are deployed to monitor and detect environmental events occurring in their vicinity. This information is then transferred through the underwater network to a hybrid node, typically a surface gateway node that uses radio waves to relay data received from an underwater acoustic network. By deploying multiple surface gateways, the number of receivers of acoustic transmission increases which allows sensor nodes to forward packets to nearby gateways directly [13,14]. Thus, all the surface-level gateways and control station may be regarded as a single virtual sink. This approach reduces the total propagation and transmission time because electromagnetic wave propagation is of orders of magnitude faster than acoustic waves and the bandwidth of radio is much wider than acoustic. Since acoustic communications consume more energy than radio communications [2], energy consumption can also be reduced. As a critical hub for underwater sensor networks and shore control centers, the surface gateway deployment is particularly essential. Another challenge that needs to be addressed is that the underwater sensor nodes are typically mobile with the water current. Therefore, deployment of surface sink nodes needs to be readjusted at a set of intervals to ensure the connectivity and coverage of the whole network. However, when the number and frequency of adjustments increases, it is bound to increase the complexity of the deployment and deviate from the original intention. Thus, it requires a design for a deployment strategy to guarantees the maximum coverage and connectivity in both time and space for underwater sensor networks.

Fundamentally, optimal deployment of multiple surface-level gateways must consider many factors such as the deployment surface area and the budget constraints that will limit the number of purchased gateways. A surface gateway deployment may also be designed to satisfy a certain set of desired network performance measures, such as life time, average end-to-end delay, coverage, and connectivity. The previous research [15] assumes *a priori* knowledge of network demand and statistical models to characterize channel status, MAC protocols, routing protocols, and mobility patterns. However, these simplifying assumptions cannot account for possible changes in the environment such as evolving mission requirements or the mobility and status of underwater nodes, eliminating static gateway deployment as a viable strategy. Another work [16] adds the idea of prediction, but the optimization algorithm and initialization are relatively simple.

This paper presents Prediction-Assisted Dynamic Surface Gateway Placement, named PADP, a dynamic surface gateway deployment scheme not only incorporating coverage, the end-to-end delay, and connectivity constraints, but also extending the time interval of each redeployment. Unlike most gateway deployment strategies that is either static done once or static but periodic where the timing of redeployment does not guarantee the full coverage of underwater nodes at all times, our PADP maximizes the average coverage, reduces end-to-end delay, controls the frequency of redeployment to reduce power consumption, ensures connectivity while satisfying budget constraints. PADP considers the mobility of underwater nodes for gateway redeployment decision by predicting their future positrons and allowing gateways relocation to prevent any coverage or connectivity loss during network lifetime compared to static/periodic deployment To achieve these objectives, PADP combines several algorithmic techniques, including the IMM (Interacting Multiple Model) for tracking, branch-and-cut to solve the underlying optimization problem, and a clustering algorithm based on the disjoint-set data structure. Compared to static deployment, the deployment in PADP is determined by considering the positions of *virtual nodes*, which consist of both currents and predicted positions of sensor nodes. By considering the essential network performance while using currents and predicted positions of sensor nodes to find the optimal surface gateway redeployment position, PADP achieves a better coverage in time and space and can significantly reduce the frequency of redeployment and the consumed energy.

The remainder of this paper is organized as follows. Related work is reviewed in Section 2. Section 3 introduces background concepts, while Section 4 describes the PADP Algorithm in detail. Simulation results are presented in Section 5. Section 6 offers conclusions and directions for future work.

## 2. Related Work

The sensing coverage problem has received significant attention in the context of wireless sensor networks [17,18,19,20,21]. Several algorithms [22,23,24] have been proposed to achieve full sensing coverage, while others [25,26] seek to maximize the lifetime of a sensor network by keeping only a subset of the sensor nodes active at any given time. The deployment problem for UWSN is considered to be a grand challenge because one must overcome additional uncertainties posed by aquatic environments. A surface gateway deployment framework to reduce end-to-end delay and energy consumption was proposed in [15]. The authors formulate the deployment problem as an integer linear programming (ILP) problem, where candidate gateway positions are known. However, they also assume prior knowledge of the channel status, MAC protocols, routing protocols, and mobility patterns, limiting its applicability to networks experiencing more realistic conditions. In [27] the authors analyzes the impact of the sensing and transmission ranges on coverage, connectivity, and network diameter. They also provide conditions on the node transmission range needed to complete the desired degree of connectivity as well as the sensing range required to achieve a desired degree of coverage in a region containing a predetermined number of nodes. In [28] the authors investigate redeployment needs when sensor nodes passively drift away from an area where sensing coverage is to be maintained. To characterize the continuing drift of sensor nodes, a random walk model is assumed. The drift model is complemented by a corrective redeployment strategy to replace lost sensors. The primary goal of [29] was to improve the collective coverage of the network by repositioning underwater sensors. Instead of merely minimizing the total cost of moving all sensors during the repositioning process, this approach also seeks to minimize the maximum cost of repositioning a set of sensors assigned to a single AUV, ensuring that AUV deplete their fuel supplies more uniformly [30]. Both [28,29] are focused on enhancing coverage by repositioning the underwater sensor nodes which is practically difficult to achieve. Adjust the depth of sensors after random deployment at the bottom of the ocean to achieve 1-coverage, which is defined as a deployment where each node is covered by at least one gateway. A central station directs the movements of each sensor after initial deployment. Thus, the approach is not fully distributed, requiring connectivity which was not explicitly addressed. Furthermore, detailed analysis of coverage performance based on their random approach was not provided. A Game Theory Field Design (GTFD) model of the deployment problem was proposed in [31]. This model is a two-player zero-sum matrix game between a network designer and an adversary vehicle, where the optimal strategy of the adversary is formulated as a linear programming problem. Solving this problem enables effective sensor node deployment. In [32] the authors proposes a distributed algorithm to improve coverage. In this approach, nodes are deployed randomly at the surface and are connected to buoys with wires. Nodes may then adjust their depths to improve coverage. However, it is unrealistically assumed that any two nodes can communicate through the buoys at the surface regardless of their transmission range. To improve coverage and provide connectivity with the surface stations, the authors [33] presented a distributed node deployment technique for UWSNs after random deployment on the seafloor. However, nodes are only capable of adjusting their depth. Depth adjustments are calculated based on a 2-dimensional projection of node coverage overlap, where graph coloring is applied to reduce the coverage problem from the 3-dimensional case. In [34] a multiple gateway deployment is proposed based on cuckoo optimization algorithm which performs periodic deployment to cope with nodes drifting. A more recent work that adaptively considers redeployment of gateways and takes into consideration the dynamics of the underwater water environment is presented in [35]. However, this work assumes a heterogeneous acoustic/optical underwater wireless sensor network and its main objective is reducing energy. As seen above, most of the redeployment strategies for underwater wireless sensor networks are concerned with redeploying underwater nodes which is practically hard due to the remote deployment locations of underwater nodes below the sea surface [15]. Therefore, gateway deployment strategies to enhance various performance metric presents a better solution. However almost all gateway deployment strategies found in the literature assume either a static network environment or deals with the dynamic case by periodically running the static deployment algorithm that negatively affects the connectivity, coverage, and energy consumption.

## 3. Background

This section provides background knowledge required to implement PADP, including the design challenges posed by UWSN deployment as well as the IMM algorithm.

### 3.1. Design Challenges

A surface gateway deployment for an UWSN must address the following major challenges:

#### 3.1.1. Long Propagation Delays

Due to the low propagation speed of acoustic signals, UWSNs suffer from long propagation delays. This significantly increases the end-to-end latency between data transmission at the sensor and reception at the base station situated in a facility onshore or on a boat. The primary goal of surface gateway deployment is to reduce this latency. Such a deployment should maximize coverage of the sensor nodes to reduce the number of relays performed by the underwater network. This will enable signals to travel shorter distances, lowering the associated delays.

#### 3.1.2. Sensor Node Mobility

In many cases, terrestrial sensor networks are immobile. However, sensor nodes in the underwater environment often exhibit passive mobility induced by water currents or proactive mobility made possible by sources such as an internal propulsion system. This mobility further complicates the deployment process, commonly requiring frequent redeployment. Therefore, sensor node mobility must be explicitly considered when deploying the gateways to preserve a desired level of coverage.

#### 3.1.3. Energy Constraints

Underwater sensor nodes are typically powered by a battery, which can be difficult or impossible to recharge. Thus, in most cases, the life time of a sensor node is restricted to a limited power supply. This power resource constraint imposes strict energy conservation requirements on the deployment strategy. Algorithms requiring frequent redeployment will not be suitable for application to UWSNs.

### 3.2. IMM

To confront the additional complexity posed by sensor node mobility PADP implements a tracking algorithm to predict the position of sensor nodes in multiple future time steps. In practice, however, sensor nodes can exhibit complicated movement patterns, indicating that a single model of mobility may be ineffective. Hence, we employ an IMM estimator to accommodate multiple patterns of maneuvering. A brief introduction to the IMM estimator is provided here. A detailed description can be found in [32].

The IMM is an adaptive estimation approach. Unlike many other methods which assume a particular node movement pattern, the IMM filter incorporates each of the possible node movement patterns, executing a set of filters corresponding to each movement pattern in parallel. The overall state estimate is then determined as a combination of the conditional estimates of the individual models.

PADP considers two node movement patterns defined as follows:

- *Uniform motion*: movement in a straight line at constant velocity, modeled by a Kalman filter.
- *Maneuver*: coordinated turn at a constant rate of turn and constant speed, modeled by an extended Kalman filter.

**Figure 1:**
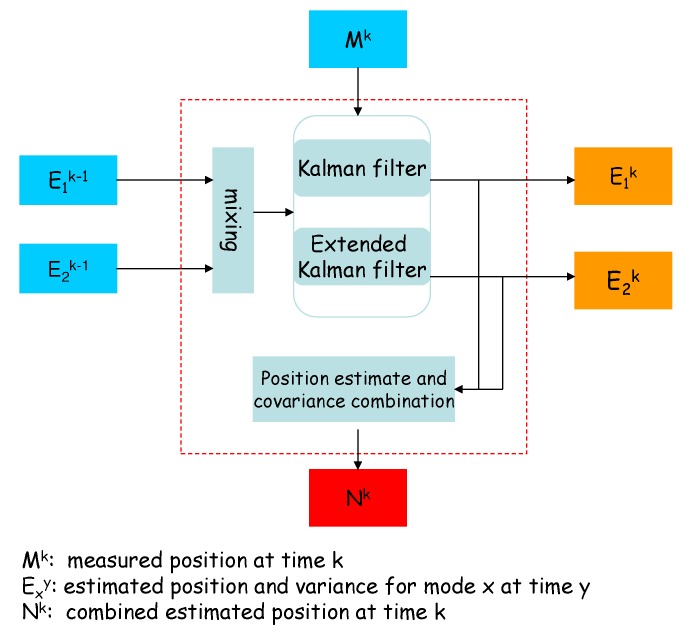
Block diagram of the tracking routine.

These two filters run in parallel. $M^{k}$ stands for the measured position at time k, $E_{x}^{k}$ is the predicted position using the paralleled filters, and $N^{k}$ is the final estimated position. The combination of the estimates produced by the two filters determines the final estimate of the node’s position as shown in Figure 1.

Given that nodes’ moving pattern, uniform motion, and maneuver, can change from one mode to another, the IMM estimate of the node location is computed as a weighted summation of the estimates from multiple mode-matching filters. The weights of the two parts are calculated by the maximum likelihood estimation method. A brief introduction on the IMM estimator is provided in the sequel, and a detailed description can be found in the book [32]. A Markov chain transition matrix is characterizing the transition probability of the node’s moving pattern from one mode to another, is introduced to update the weighting coefficients. Except that the initial condition of each filter is a combination of the model-conditioned estimates of the multiple filters at the preceding state, operation of each filter is identical to that of the Kalman filter or the extended Kalman filter, depending on whether the state equation under the considered mode is linear or not.

## 4. PADP Algorithm Description

This section introduces the details of PADP, including network architecture, problem statement, assumptions, and formulation.

### 4.1. Network Architecture

**Figure 2:**
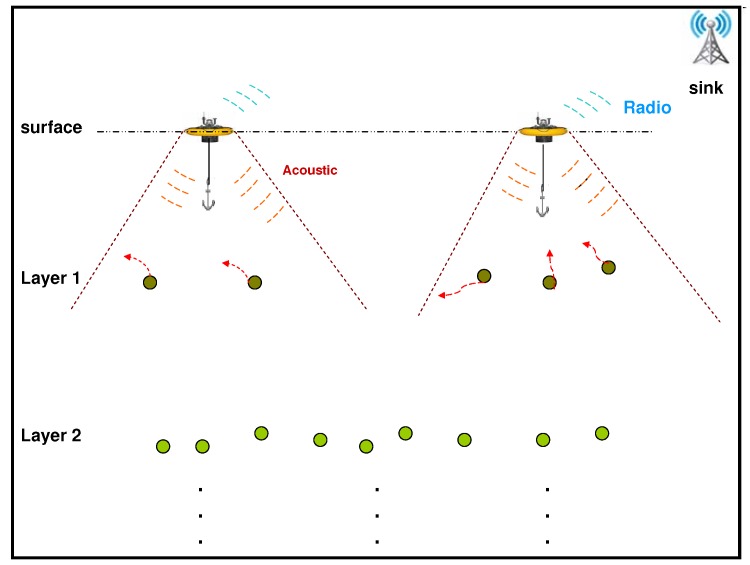
Network architecture.

We consider the hierarchical underwater sensor network architecture shown in Figure 2. Lower layer sensor nodes collect sensed information and send them to surface gateway through middle layer relay sensor nodes. The gateways are responsible for transmitting the information back to a data center usually located onshore.. Each sensor node will be equipped with a payload, so that it will stay in a certain depth of the water. The hierarchical structure is formed with different kinds of payload. (Please note that we only consider sensor nodes in layer 1 in this work because we focus on surface gateway deployment.)

Such a network consists of three types of nodes:

- *Sinks*: control stations on the shore or aboard a boat, directly connected to all surface gateways through radio communication.
- *Surface gateways*: hybrid nodes associated with buoys, which communicate with underwater sensor nodes using acoustic communication and sinks via radio communication.
- *Underwater sensor nodes*: ordinary sensor nodes organized in layers at various depths to collect environmental information and forward it to surface gateways.

### 4.2. Problem Statement

To maximize the utility of surface gateways in the network topology of Figure 2, coverage of the underwater sensor nodes should be maximized so that sensor nodes can forward data packets to the nearest gateway. The deployment problem is characterized as an optimization problem to identify the best locations for a fixed number of gateways to achieve maximum coverage. Please note that our proposed algorithm is based on centralized pre-deployment optimization, the gateways obtain all the information from the sensor nodes and make all the decisions.

Due to the sensor node’s passive movement, coverage continually changes in the underwater environment (Please note that in this work, the passive movement is considered to be the main reason for coverage change. Other environment changes like transmission range which has an impact on coverage are put to future work). Therefore, the coverage achieved by a gateway deployment based on the present sensor locations may experience degradation in coverage over a period of time. In light of this, a practical technique to place the gateways is to predict the position of each sensor node at a sequence of time points and treat each position as a single node, referred to as a *virtual node*. By identifying the maximum coverage of these virtual nodes, node coverage can be maximized throughout the period.

**Figure 3:**
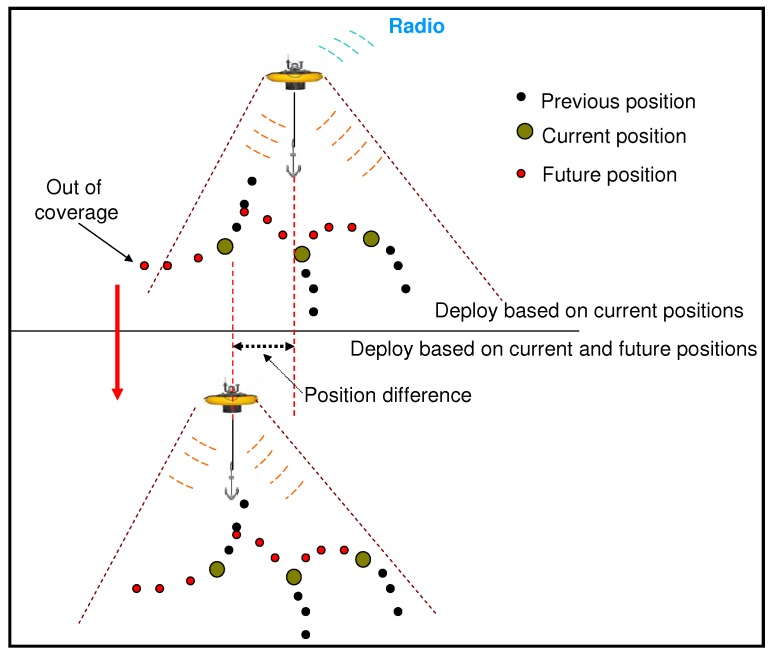
Naive deployment (**top**) predictive deployment (**bottom**).

The upper portion of Figure 3 shows a naive deployment based on the present sensor node locations, denoted as large green dots. Black dots represent previous positions, and red dots indicate future positions, as predicted by the IMM. The figure illustrates that some of the sensor nodes move out of range of the gateways after several (three) predicted time steps, degrading coverage. The lower portion of Figure 3 indicates a gateway deployment based on both the present and future sensor node positions. Hence, the sensor nodes remain within range during the entirety of the predicted time interval, preserving a high level of coverage.

### 4.3. Assumptions

The following assumptions are made:

- The position of each gateway is known after deployment. And an underwater node can always know where it is [36,37,38].
- The studied underwater sensor nodes moving patterns are relative moving patterns to surface gateway.
- Underwater sensor nodes are organized in a layered structure and only nodes in Layer 1 can communicate with surface gateways.
- Localization and synchronization services are available so that an underwater sensor node is always able to estimate its current location.
- Gateways and sensor nodes share a common transmission range *d*.

### 4.4. Problem Formulation

This section formulates the gateway deployment problem as a linear optimization problem, which can be solved with the branch-and-cut method. The branch-and-cut method is widely used for solving mixed integer programming (MIP) problems. In underwater sensor networks, the number of transport nodes and water gateway nodes in the first layer is limited, so we use the branch-and-cut method because of its low computational complexity.

#### 4.4.1. Definition

The current and future positions of sensor node *i* are denoted $p_{i}=\left[{p_{i}}^{0},{p_{i}}^{1},{p_{i}}^{2}\ldots ,{p_{i}}^{n}\right]$, where ${p_{i}}^{0}$ is the current position of node *i*, as determined by the localization service. The future positions ${p_{i}}^{1},{p_{i}}^{2}\ldots ,{p_{i}}^{n}$ are predicted by the IMM described in Section 3. We consider a deployment of *m* sensor nodes in Layer 1, where the position of each sensor node at Layer 1 is predicted for *n* time slots into the future. Furthermore,

(1) $$Y_{iqk}=\left\{\begin{array}{cc} 1 & \mathrm{node}i\mathrm{covered}\mathrm{by}\mathrm{gateway}q\mathrm{at}\mathrm{time}k\\ 0 & \mathrm{otherwise} \end{array}\right.$$

Since the transmission range of gateways and sensor nodes is a fixed constant *d*, gateway coverage of a sensor node may be expressed as:

(2) $$\begin{array}{c} \|C_{q}-p_{i}^{k}\|\cdot Y_{iqk}\leq d \end{array}$$

where $C_{q}$ is the position where gateway *q* is deployed. The values of $C_{q}$ must be identified by the optimization procedure. If the distance between $C_{q}$ and $p_{i}^{k}$ exceeds *d* then $Y_{iqk}$ will be 0 according to Equation (2). If the distance is less than *d* then $Y_{iqk}$ can either be 0 or 1. However, maximization of $Y_{iqk}$ in the objective function presented below will ensure that the value is 1 more often than 0. Thus, matrix $Y_{iqk}$ provides a simple way to determine if a sensor node is covered.

#### 4.4.2. Objective Function 1: Coverage

Equation (3) is the objective function to maximize coverage. PADP defines coverage as the number of times sensor nodes are covered by the deployed gateways during the *n* future time points. Hence, effective placement of the gateways should cover as many of the *m* sensor nodes at Layer 1 during the *n* time steps. It is equivalent maximizing the coverage of the *virtual nodes* introduced in Figure 3.

(3) $$\begin{array}{c} \max_{C_{q}}(\sum_{k=0}^{n}\sum_{i=0}^{m}\max_{q}Y_{iqk}), \end{array}$$

#### 4.4.3. Constraints

While coverage is the primary focus of PADP, a total of four constraints are imposed. These constraints include a budget limit for gateways, an energy constraint for redeployment, as well as minimum coverage and connectivity.

- Budget limit: constrains the number of available gateways. It is assumed that the budget is pre-defined and does not change. Hence, the number of gateways will not increase during the optimization procedure. Considering the budget permits *N* gateways:
  (4) $$\begin{array}{c} 1\leq q\leq N \end{array}$$
- Maximum allowable travel distance: PADP assumes that gateways are relatively static locations for the duration of a deployment. However, in each redeployment, gateways can actively move to a position where they are needed. For this purpose, each surface gateway *q* possesses a maximum allowable travel distance for redeployment. This constraint is imposed to conserve power consumption. Because each gateway assumes an initial position and must expend energy to relocate to new positions it is assumed that a gateway is aware of its battery so that it can reduce the power needed to return to shore or a boat to be recharged. The maximum allowable travel distance for redeployment is denoted $\lambda_{q}$, while $C_{q}^{0}$ is the initial position of gateway *q*, so that:
  (5) $$\begin{array}{c} \|C_{q}-C_{q}^{0}\|\leq \lambda_{q},\forall q. \end{array}$$
- Minimum coverage: considers the virtual nodes of Figure 3, these are not real sensor nodes, i.e., only *m* sensor nodes exist at any specific time. To ensure the desired number of sensor nodes are covered at any time, a minimum coverage γ is assigned, so that:
  (6) $$\begin{array}{c} \sum_{i=0}^{m}\max_{q}Y_{iqk}\geq \gamma ,\forall k \end{array}$$
- Connectivity constraint: sensor nodes in Layer 1 are clustered during surface gateway deployment to guarantee the connectivity of these sensor nodes. A cluster consists of a group of connected sensor nodes, but members of a cluster are not connected to the sensor nodes of any other cluster. Connectivity requires that there must be at least one sensor node in each cluster covered by the surface gateways. Letting ϕ(υ) represent the cluster υ, this constraint may be expressed as:
  (7) $$\begin{array}{c} \sum_{i\in \phi (\upsilon )}\sum_{q}Y_{iqk}\geq 1,\forall k,\upsilon \end{array}$$

### 4.5. Clustering Algorithm

In the process of optimizing, it is certain that the sensor nodes in one layer are not directly connected to the surface nodes, instead they can be connected by relay. So, we need to consider the connectivity between nodes. The disjoint-set data structure partitions sensor nodes in Layer 1 into disjoint sets referred to as *clusters* according to their locations and transmission range *d*. The Aalgorithm 1 uses three basic functions. MAKE-CLUSTER(*x*) creates a new cluster with a single member *x*. This operation requires that *x* is not already in any other cluster. UNION(*x*, *y*) unites the clusters containing *x* and *y* into a new cluster. FIND-CLUSTER(*x*) returns a pointer to the representative of the (unique) cluster containing *x*. The scheme requires O(nlog(n)) time, where *n* stands for the number of nodes.

**Algorithm 1** Operations of disjoint-set data structure.Definitions: G: the entire set of sensor nodes E[G]: the cluster of directly connected sensor nodes p[x]: parent of node x rank[x]: rank of node x (the term rank is used instead of depth since it stops being equal to the depth if path compression (described below) is also used.) Clustering algorithm: for each node i ∈ G  do MAKE-CLUSTER(i) for each edge (i, j) ∈ E[G]  do if FIND-CLUSTER(i) ≠ FIND-CLUSTER(j)  then UNION(i, j) MAKE-CLUSTER(x): p[x] ⟵ x rank[x] ⟵ 0 UNION(x, y): LINK(FIND-CLUSTER(x), FIND-CLUSTER(y)) LINK(x, y): if rank[x]>rank[y]  then p[y] ⟵ x  else p[x] ⟵ y if rank[x] = rank[y]  then rank[y] ⟵ rank[y] + 1 FIND-CLUSTER(x) with path compression: if x ≠ p[x]  then p[x] ⟵ FIND-CLUSTER(p[x]) return p[x]

### 4.6. Linearization

Please note that Equations (2), (3) and (6) are nonlinear and need to be converted to linear form if they are to be solved with branch-and-cut. For Equations (3) and (6), a new variable *X* is introduced:

(8) $$X_{ik}=\left\{\begin{array}{cc} 1 & \mathrm{sensor}\;\mathrm{node}\;i\;\mathrm{covered}\;\mathrm{at}\;\mathrm{time}\;k\\ 0 & \mathrm{otherwise} \end{array}\right.$$

The relationship between *Y* and *X* is:

(9) $$\begin{array}{c} X_{ik}<=\sum_{q=0}^{N}Y_{iqk}, \end{array}$$

where *N* is the number of available gateways. The objective function becomes:

(10) $$\begin{array}{c} \max_{C_{q}}\left(\sum_{k=0}^{n}\sum_{i=0}^{m}X_{ik}\right), \end{array}$$

while Equation (6) becomes:

(11) $$\begin{array}{c} \sum_{i=0}^{m}X_{ik}\geq \gamma ,\forall k. \end{array}$$

For Equation (2), a dependent binary variable *Z* is introduced for each *Y*, defined as:

(12) $$\begin{array}{c} Y\leq M\cdot (1-Z) \end{array}$$

where *M* is a large positive number and *Z* is:

(13) $$Z_{iqk}=\left\{\begin{array}{cc} 1 & \mathrm{for}\;\|C_{q}-p_{i}^{k}\|>d\\ 0 & \mathrm{for}\;\|C_{q}-p_{i}^{k}\|\leq d \end{array}\right.$$

The following constraint may be added to characterize this relationship:

(14) $$\begin{array}{c} Z_{iqk}\geq \left(\right\|C_{q}-p_{i}^{k}\left\|-d\right)/M \end{array}$$

Please note that when $\|C_{q}-p_{i}^{k}\|>d$, the right-hand side is decimal-valued in the interval (0,1), and *Z* will be fixed at 1. If $\|C_{q}-p_{i}^{k}\|=d$ then the right-hand side is 0, whereas $\|C_{q}-p_{i}^{k}\|<d$ makes the right-hand side negative. In either of these last two cases, the constraint is redundant because *Z* can be either 0 or 1 and is therefore not restricted.

Based on the constraint in Equation (12), it follows that if $\|C_{q}-p_{i}^{k}\|>d$ then Z=1 and *Y* is fixed at 0. Similarly, if $\|C_{q}-p_{i}^{k}\|\leq d$ then Z=0, so *Y* is not fixed. However, maximization will tend to make Y=1. This recharacterization of the optimization procedure renders all the constraints and objective function linear, enabling the solution by the branch-and-cut method.

### 4.7. Objective Function 2: Reduce Average End-To-End Delay

End-to-end delay is another critical performance measure in UWSNs. Therefore, a second objective function is introduced. This objective function must also satisfy the constraints given above. End-to-end delay is the sum of the per-hop delays over the entire path from the source to destination, where packets are respectively generated and received. There are three primary types of delay: queuing and channel access delays, transmission delay, and propagation delay. Since queuing and channel access delay are more related to MAC protocol, which is beyond the scope of this work, we mainly consider the transmission and propagation delay. Due to the low bandwidth and propagation speed in UWSNs, the transmission and propagation delays are much longer compared to wireless sensor network on the ground.

To reduce the average end-to-end delay, which may be computed as the average end-to-end latency for each packet from its source to destination, a gateway deployment should cover sensor nodes in Layer 1 that commonly experiences high traffic as shown in Figure 2. This approach will reduce the number of hops for a large proportion of packets, subsequently decreasing the average end-to-end delay. The rationale behind this approach is that one cannot be certain of the underlying sensor node topology. Some sensor nodes in the first layer may have more connections to sensor nodes in a lower layer than others. Layer 1 nodes with a larger number of connections are more likely to experience higher volumes of traffic. Therefore, covering sensor nodes experiencing high traffic will reduce the average end-to-end delay of all packets.

PADP monitors traffic volumes during the previous ψ seconds and assigns a weight of ω, to each sensor node *i* in Layer 1:

$$\omega_{i}=\frac{\eta_{i}}{\sum_{k=1}^{m}\eta_{k}}$$

where $\eta_{i}$ represents the number of packets received by sensor node *i* during the last ψ seconds.

The objective function to minimize average end-to-end delay is:

(15) $$\begin{array}{c} \max_{C_{q}}\left(\sum_{k=0}^{n}\sum_{i=0}^{m}\max_{q}\omega_{i}Y_{iqk}\right), \end{array}$$

Combine the above two equations, the linearization process is similar to the 4.6 Linearization, so it can be referred to the method above to achieve the optimization for reducing average end-to-end delay. In addition, we can solve this optimization problem with no changes to the constraints generates a solution for the deployment with reduced the average end-to-end delay.

### 4.8. Initial Deployment

**Figure 4:**
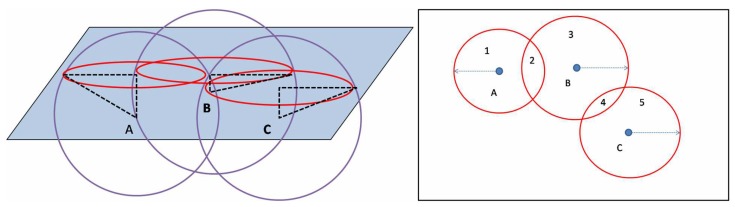
Initial deployment algorithm.

We calculate the initial deployment is referring to the scheme in the paper [39]. In Figure 4, the tangent plane of the communication ball of an underwater node and surface of the water can be regarded as some surface disks. In addition, these intersecting disks can divide the water surface into several logical regions which got the order depending on the level of coverage it is covered. We select the high order logical regions centroid will be the initial position of the surface gateway node.

## 5. Performance Evaluation

### 5.1. Simulation Settings

Simulations were conducted in MATLAB, while branch-and-cut was implemented in CPLEX to solve the optimization problem. In the experiments, 50 sensor nodes were deployed in Layer 1. All sensor nodes predict their future positions for *N* seconds with the IMM algorithm. The statistics are obtained from 100 runs. To guarantees the maximum coverage and connectivity in both time and space for underwater sensor networks. We consider the dynamic of the sensors, the dynamic means that the underwater sensor nodes are typically mobile with the water current. So, in simulation, we consider dynamic deployment rather than static which is inconsistent with the real underwater environment. To ensure a fair comparison, we performed an entirely controlled simulation to make sure at the beginning of each round simulation, the positions of sensor nodes and surface gateways are the same. The parameters of the simulation are shown in Table 1.

The mobility behavior of the sensor nodes is characterized by the kinematic model [40], in which the node velocities are:

(16) $$\left\{\begin{array}{c} V_{x}=k_{1}\lambda v\sin\left(k_{2}x\right)\cos\left(k_{3}y\right)+k_{1}\lambda \cos\left(2k_{1}t\right)+k_{4}\\ V_{y}=-\lambda v\cos\left(k_{2}x\right)\sin\left(k_{3}y\right)+k_{5} \end{array}\right.$$

where $V_{x}$ and $V_{y}$ represent the velocity along the *x* and *y* axis, respectively. $k_{1}$, $k_{2}$, $k_{3}$, and λ,v are variables related to environmental factors such as tides and bathymetry. These parameters will vary in different settings. Random variables $k_{4}$ and $k_{5}$ represent additional random physical factors.

The queuing and channel access delays are assumed to be constant and represented as $D_{q}$ and $D_{c}$ to estimate delays. The bit rate ($T_{r}$) of 3.1 kbps is based on our OFDM modem, and the ranging message ($P_{r}$) is 40 bytes, which makes the transmission delay 0.103 s. The propagation speed is 1500 m/s. In ocean, a maximum random speed of nodes under different mobility models is usually set to 5 m/s on a random direction. A 6 s interval was selected for nodes to predict their future position. It will give a limit on the maximum traveled distance of 30 m. This distance can affect the reachability of gateways by the underwater node and can be a reasonable deciding factor for gateway redeployment.

### 5.2. Results and Analysis

#### 5.2.1. Dynamic vs. Static

**Figure 5:**
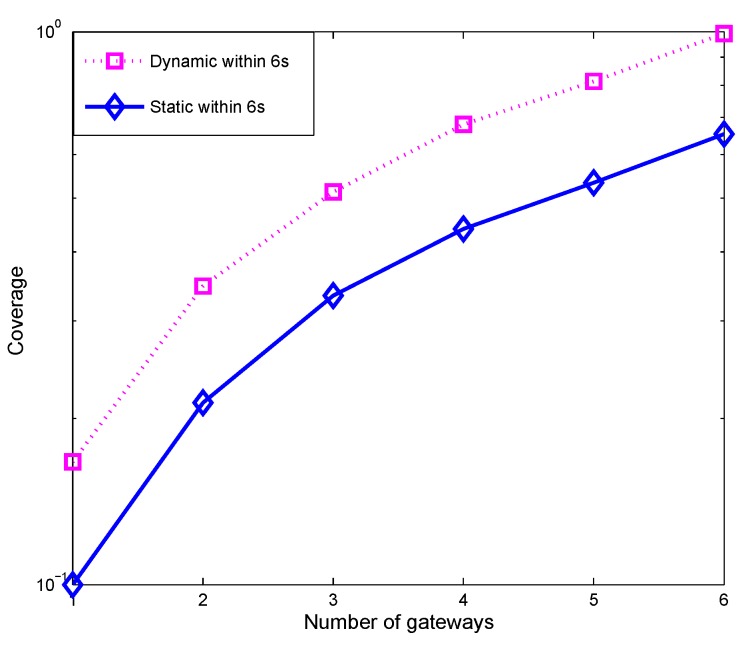
Dynamic vs. Static deployment.

Figure 5 shows that dynamic deployment outperforms static deployment with respect to coverage, defined as the ratio between the nodes covered and the total number of nodes. Comparing dynamic and static deployment over six seconds intervals reveals that dynamic deployment attains superior coverage because PADP considers sensor node mobility. Furthermore, the deployment was determined based on the predicted positions of sensor nodes not just their present locations. This additional consideration preserves higher levels of coverage for more extended periods. The figure also illustrates that for both dynamic and static deployment, the coverage achieved is higher for the experiments with more gateways deployed.

#### 5.2.2. Impact of Transmission Range

**Figure 6:**
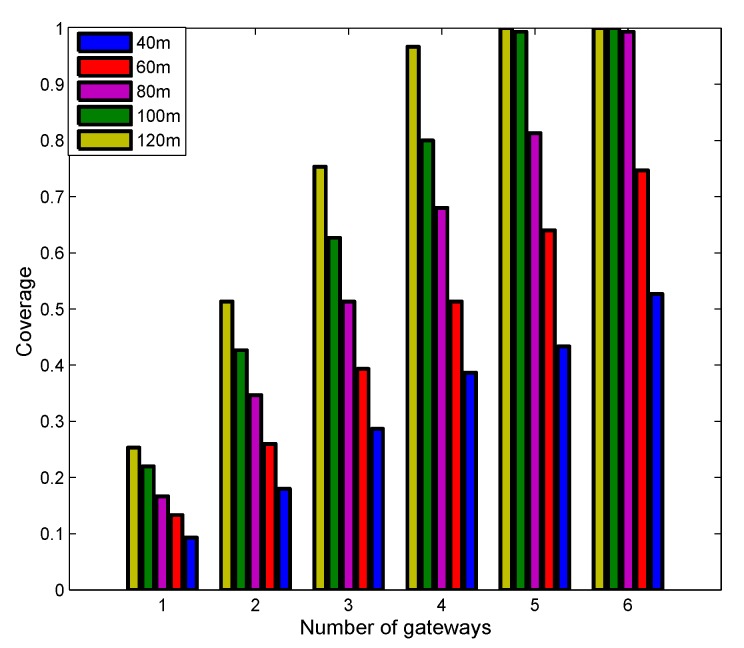
Impact of transmission range.

Figure 6 demonstrates the impact of transmission range on coverage. It is easy to see that increasing transmission range improves coverage. From this point of view, it is always helpful to have a long transmission range. However, the transmission range is related to the transmission power [41]. In some cases, increasing transmission power will increase the transmission range, but energy conservation is critical for underwater sensor nodes because they are powered by the battery and are difficult to recharge. Thus, a natural tradeoff between coverage and energy consumption exists. In some scenarios more power means more reflections and hence more difficulties for the modem to correctly decode the message. In this case, by reducing the transmission power, the transmission range can be increased.

#### 5.2.3. Impact of Minimum Coverage

**Figure 7:**
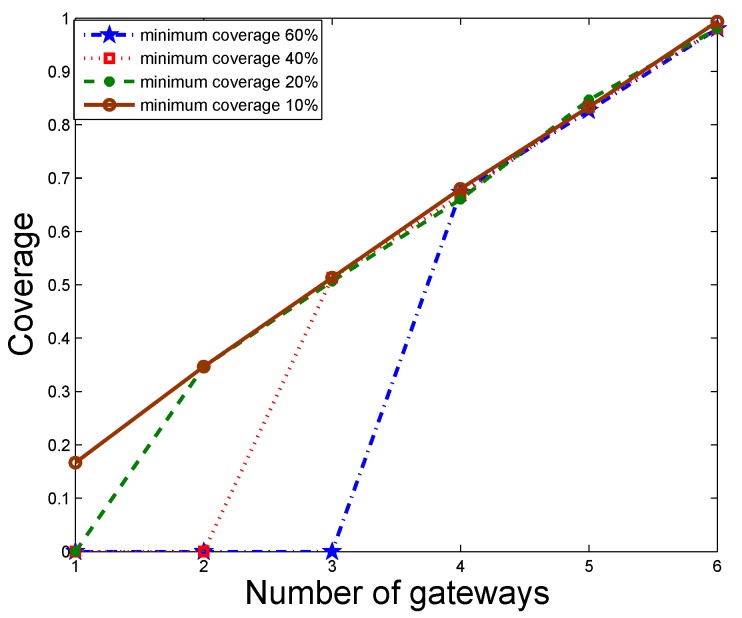
Impact of minimum coverage.

Figure 7 shows the impact of imposing a minimum level of coverage, which in some cases can cause the optimization problem to lack a feasible solution. For instance, when the minimum coverage required is 60%, a deployment must use at least three gateways. It can also be seen that after the minimum coverage is satisfied, the constraint will no longer be an issue and solutions to the deployment can be found without difficulty.

#### 5.2.4. Impact of Maximum Travel Distance

**Figure 8:**
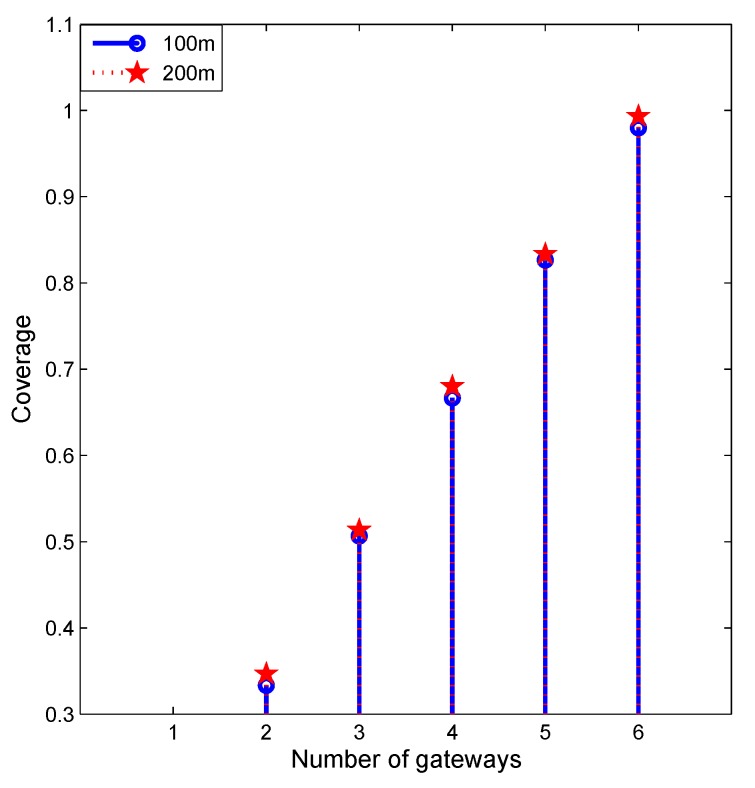
Impact of maximum travel distance.

Figure 8 reveals the impact of the maximum allowable travel distance on coverage. When the maximum allowable travel distance reflexes, the coverage gets increased. That is because it prevents the gateways from moving too far from their original positions, and that will constrain the deployment. Therefore, when it relaxes, the coverage will increase.

#### 5.2.5. Impact of Tracking Accuracy

**Figure 9:**
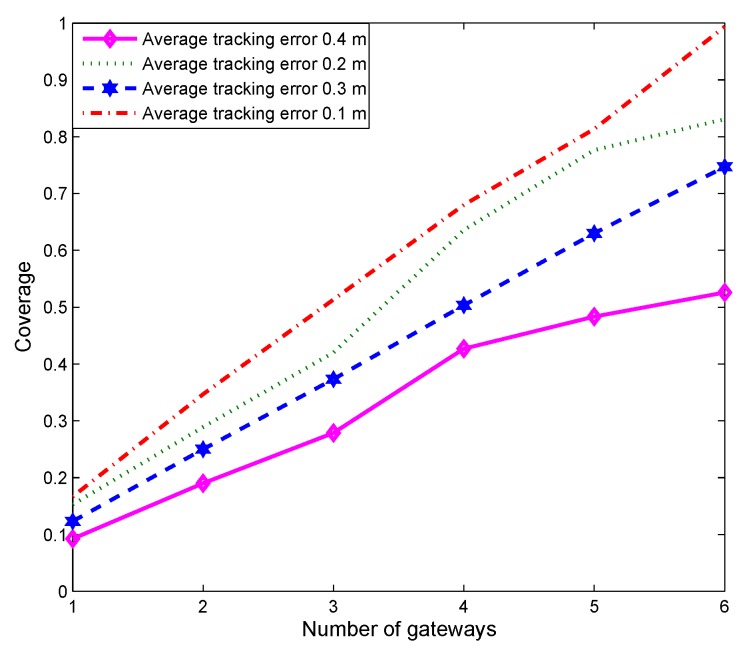
Impact of tracking accuracy.

Figure 9 illustrates the impact of tracking accuracy, defined as the precision of the predicted positions of the sensor nodes. Tracking accuracy cannot be controlled because the IMM framework computes the locations. Thus, to quantify accuracy, we use the real positions unknown to the IMM in addition to the accuracies specified in the figure. In IMM framework, the last estimation result will be added to each prediction cycle, and the tracking accuracy will be greatly reduced with the increase of speed and the extension of prediction time. Since the predicted positions directly affect the quality of a deployment, improved tracking accuracy results in better coverage.

#### 5.2.6. Minimize Average End-To-End Delay

**Figure 10:**
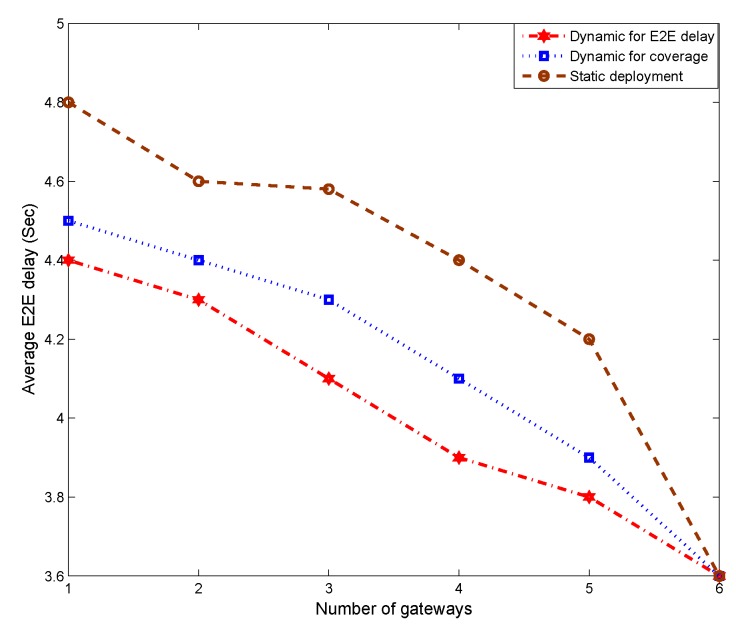
Minimization of average end-to-end delay.

Figure 10 shows the average end-to-end delay under different objective functions. “E2E delay” identifies the solution that minimizes the average end-to-end delay, while “coverage” corresponds to the solution that maximizes coverage. “Static” deployment only considers the current sensor node positions and traffic. The results indicate that end-to-end delay is lowest when the objective to minimize it is explicitly invoked. However, the solutions to the end-to-end and coverage objectives are equal when the number of gateways is six because this number of gateways is sufficient to cover all sensor nodes in Layer 1. Thus, there is no difference in the two solutions concerning the average end-to-end delay.

#### 5.2.7. Impact of Monitoring Interval

**Figure 11:**
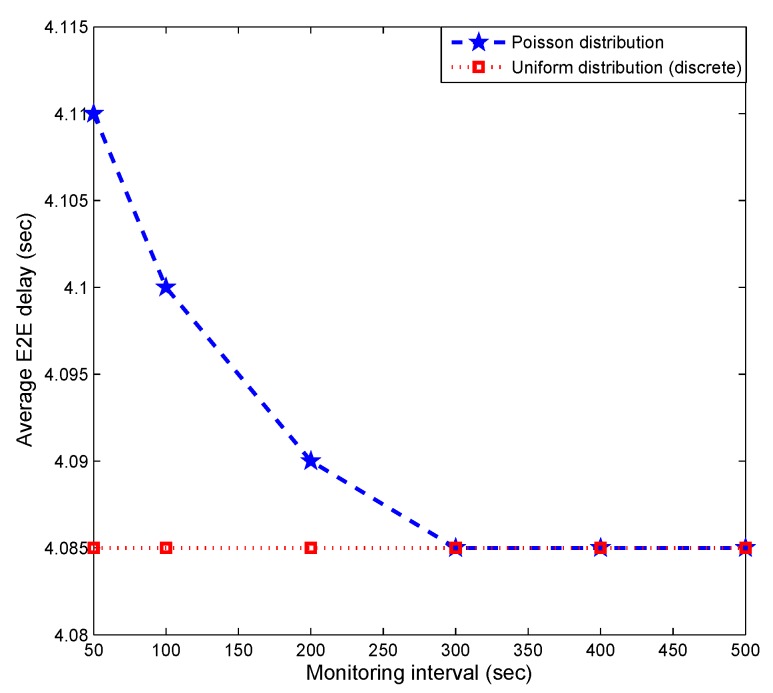
Impact of monitoring interval.

Figure 11 explores the impact of the monitoring interval ψ during which traffic volume counts are collected for each sensor node in Layer 1. Increasing ψ enables more accurate determination of the relative amount of traffic on each of the sensor nodes in Layer 1, thus reducing delay. However, the end-to-end delay is invariant under the uniform distribution, as one would expect.

#### 5.2.8. Impact of Mobility Pattern

**Figure 12:**
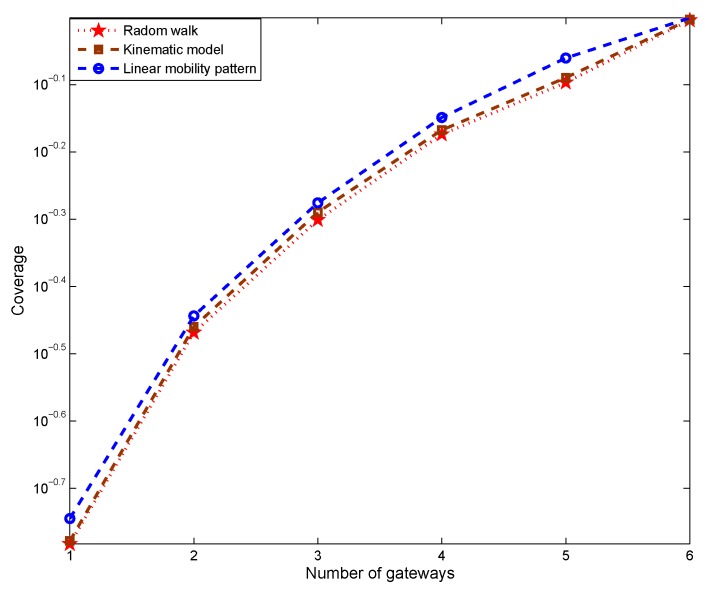
Impact of mobility pattern on coverage.

**Figure 13:**
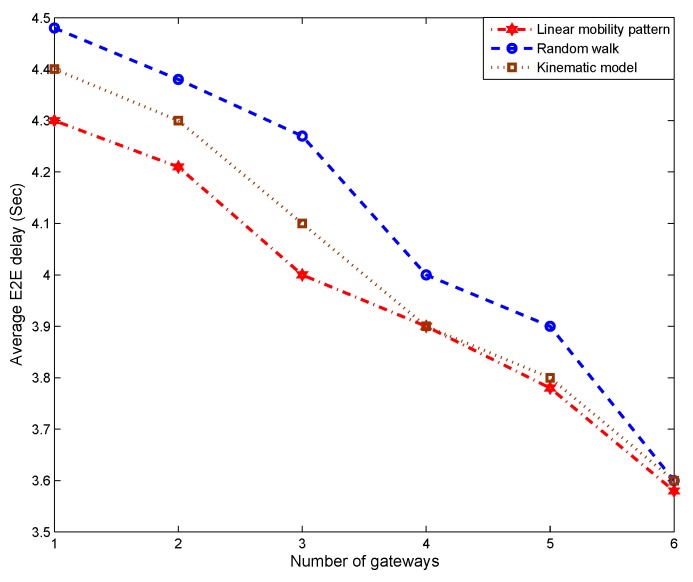
Impact of Mobility pattern on end-to-end delay.

Figure 12 and Figure 13 study the impact of the underlying mobility pattern on coverage and average end-to-end delay, where the linear mobility pattern is defined as:

(17) $$\left\{\begin{array}{c} V_{x}=k_{1}t+k_{4}\\ V_{y}=k_{2}t+k_{5} \end{array}\right.$$

Here, $V_{x}$ and $V_{y}$ represents the velocity of a sensor node along the *x* and *y* axis, respectively. $k_{1}$ and $k_{2}$ are parameters characterizing environmental factors such as tides and bathymetry. $k_{4}$, and $k_{5}$ simulate additional random factors such as rates of drift. For the purpose of simulation, $k_{1}$ and $k_{2}$ are normal random variables with mean π and variance 0.1π, while $k_{4}$ and $k_{5}$ are normally distributed with mean 0.1 and variance 0.01. Whenever the velocities reach a value greater than 5 m/s, the sign of $k_{1}$ and $k_{2}$ are reversed to become negative. When the velocities reach zero, $k_{1}$ and $k_{2}$ will change, becoming positive. The sign reversal occurs to simulate the movement of tidal.

IMM is applied to track the moving of sensor nodes, in this simulation, the same IMM model is used to track sensor nodes with three different moving patterns, including random walk, which is less predictable, to show the impact from different tracking accuracy. The results reveal that the linear mobility model enables better performance than the kinematic model in Equation (16) and random walk in terms of coverage and end-to-end delay because the IMM predicts the linear pattern more easily, enabling better tracking. Thus, the future node positions are predicted more precisely, resulting in better performance. Despite the complexity of the kinematic model and total randomness of the random walk approach that affect the accuracy of predicted positions, the IMM performs very well in these nonlinear cases. Thus, PADP achieves performance comparable to the linear mobility model despite the introduction of these more sophisticated patterns of sensor node mobility.

## 6. Conclusions and Future Work

This paper presents PADP, a surface gateway deployment scheme designed to overcome the unique challenges posed by underwater sensor networks, especially sensor node mobility. The implementation combines the Interacting Multiple Model, Branch-and-cut, and Disjoint-set data structure to ensure a high level of sensor node coverage by surface gateways, while simultaneously satisfying budget constraints, enhancing connectivity, and lowering average end-to-end delay as well as power consumption during redeployment. Thus, PADP mitigates the previously unaddressed issues of long propagation delay and energy consumption by effectively positioning gateways to relay information to a control station.

The critical advantage of PADP is in that it considers both current and predicted positions of mobile sensor nodes for gateway redeployment decision. Therefore, gateways cover more sensor nodes for longer times compared with the previous static deployment algorithm. Ensuring coverage for more extended periods will reduce the frequency and hence the cost required to readjust the buoys. Moreover, the network architecture is optimized as in [42,43] which improves the computing power and allow the network to adapt to more applications.

Future research will examine the impact of the deployment scheme on the design of network protocols, including MAC and routing, by evaluating performance measures such as end-to-end delay, throughput, and energy consumption. According to the existing research, we infer that the accuracy of prediction is proportional to the tracking accuracy. The accuracy of the IMM framework will decrease with the increase of the prediction intervals and the maximum speed of nodes. At the same time, we expect that the cycle time of gateway adjustment and update will be shorter. The tracking accuracy is a dynamic value in a dynamic network and is affected by the parameters of the networks, such as mobility model of nodes in network. We will further explore incorporating AUVs with controlled mobility into our scenario. 

References

## Figures and Tables

**Table 1:** Simulation Settings.

Parameter	Value	Parameter	Value
*d*	80 m	$\lambda_{d}$	200 m
*N*	6 s	γ	5
$k_{1}$	N(π,0.1π)	$k_{2}$	N(π,0.1π)
$k_{3}$	N(2π,0.2π)	$k_{4}$	N(1,0.1)
$k_{5}$	N(1,0.1)	λ	N(3,0.3)
*v*	N(1,0.1) m/s	*M*	100,000
$D_{q}$	0.1 s	$D_{c}$	0.1 s
$T_{r}$	3.1 kbps	$P_{r}$	40 bytes
